# Non-matrix Matched Glass Disk Calibration Standards Improve XRF Micronutrient Analysis of Wheat Grain across Five Laboratories in India

**DOI:** 10.3389/fpls.2016.00784

**Published:** 2016-06-08

**Authors:** Georgia E. Guild, James C. R. Stangoulis

**Affiliations:** School of Biological Sciences, Flinders UniversityBedford Park, SA, Australia

**Keywords:** XRF, EDXRF, biofortification, micronutrient, plant

## Abstract

Within the HarvestPlus program there are many collaborators currently using X-Ray Fluorescence (XRF) spectroscopy to measure Fe and Zn in their target crops. In India, five HarvestPlus wheat collaborators have laboratories that conduct this analysis and their throughput has increased significantly. The benefits of using XRF are its ease of use, minimal sample preparation and high throughput analysis. The lack of commercially available calibration standards has led to a need for alternative calibration arrangements for many of the instruments. Consequently, the majority of instruments have either been installed with an electronic transfer of an original grain calibration set developed by a preferred lab, or a locally supplied calibration. Unfortunately, neither of these methods has been entirely successful. The electronic transfer is unable to account for small variations between the instruments, whereas the use of a locally provided calibration set is heavily reliant on the accuracy of the reference analysis method, which is particularly difficult to achieve when analyzing low levels of micronutrient. Consequently, we have developed a calibration method that uses non-matrix matched glass disks. Here we present the validation of this method and show this calibration approach can improve the reproducibility and accuracy of whole grain wheat analysis on 5 different XRF instruments across the HarvestPlus breeding program.

## Introduction

Micronutrient malnutrition is a serious problem in developing countries due to the high dependency on staple food crops, which are often low in Fe and Zn. Staples, including wheat, rice, beans and maize, can account for up to 60% of the daily calorie intake (Cakmak et al., 2004), but contain very low levels of essential micronutrients, with Fe and Zn ranging from 5 to 150 mg kg^−1^ (Pfeiffer and McClafferty, 2007a). Furthermore, only a small fraction of micronutrients present in these crops is thought to be bioavailable (Bouis and Welch, 2010). Consequently a major focus of the HarvestPlus program is to increase the micronutrient content in these staple food crops in order to combat micronutrient malnutrition (Nestel et al., 2006; Pfeiffer and McClafferty, 2007a,b; Velu et al., 2011). This process is termed biofortification and is the process by which the nutrient density of staple crops is increased by plant breeding, biotechnology and agronomic approaches (Cakmak, 2007; Pfeiffer and McClafferty, 2007b).

Energy dispersive x-ray fluorescence (EDXRF) has been employed for the analysis of various plant materials including wheat grain (Paltridge et al., 2012a), rice grain (Paltridge et al., 2012b; Teixeira et al., 2012), pearl millet grain (Paltridge et al., 2012b), coffee (Tezotto et al., 2013), medicinal plants (Queralt et al., 2005), sugar cane (Guerra et al., 2014), and tree leaves/needles (Stikans et al., 1998). The majority of plant analysis reported with XRF requires the sample to be ground and the powder pressed in order to diminish sample heterogeneity, commonly regarded as one of the largest sources of error with EDXRF analyses (Blank and Eksperiandova, 1998; Injuk et al., 2006). However, we have illustrated that analysis of whole grains is possible when screening for Zn and Fe in biofortification programs (Paltridge et al., 2012a,b). Grinding the samples would improve reproducibility and accuracy; however this process would also reduce laboratory throughput and potentially lead to contamination during the grinding process. Consequently, the EDXRF methods developed are calibrated for analysis of whole grain samples such as wheat, rice and pearl millet. This method has been particularly successful for wheat, with tens of thousands of samples screened for Zn and Fe concentrations since 2012 (Velu et al., 2012; Guzmán et al., 2014; Hao et al., 2014; Srinivasa et al., 2014; Vishwakarma et al., 2014).

XRF analysis is strongly influenced by matrix effects (Skoog et al., 2007). Consequently, when establishing an XRF calibration using the empirical method, it is important to have a suitable calibration set comprising matrix matched samples spanning a wide range of concentrations (in this case, for Fe and Zn), with robust reference values. Due to the lack of suitable commercially available samples for use as a wheat calibration set, whole grain wheat samples acquired from a biofortification breeding program at CIMMYT, Mexico have been used (Paltridge et al., 2012a). These samples were analyzed in duplicate with ICP-OES (Wheal et al., 2011) to determine robust reference values and used for the previously reported XRF wheat calibration (Paltridge et al., 2012a). Ideally, a similar set of whole grain wheat calibration samples (with similarly robust reference values) would be used to calibrate each of the XRF instruments. Unfortunately, due to the lack of sufficient quantities of the calibration samples and strict quarantine requirements when sending plant material internationally, it is not easy to calibrate each of the XRF instruments in the HarvestPlus program with this calibration set. Instead, the calibration method file developed on the Flinders University XRF unit (with whole grain wheat samples) has been transferred electronically to most of the XRFs within the HarvestPlus wheat program. Alternatively, a calibration set of wheat samples with a validated range of Fe and Zn levels are provided by the host institution for use as a local calibration set. This requires the samples to be analyzed in a lab that provides high quality analysis and this is not always available. Both of these approaches can therefore lead to errors in the resulting XRF calibration. The electronic transfer approach is unable to account for small differences between the instruments and can lead to inaccuracies in the analysis. While in a local calibration set, the accuracy of the resulting XRF calibration is highly dependent on the quality of the reference analysis. Furthermore, the low levels of Zn and Fe in wheat along with potential soil contamination during harvesting (Yasmin et al., 2014) and sample preparation (Cubadda et al., 2001) often results in poor quality reference values that can lead to XRF calibration errors. Whilst it is evident that these errors may affect the accuracy of Fe and Zn levels reported, the resulting calibration methods are still suitable when using XRF as a screening tool in plant breeding. However, when comparing micronutrient levels in crops from different sites and on different instruments (i.e., GxE testing), these errors can result in significant differences between the results. In order to address these issues, we have investigated the use of non-matrix matched glass disks to calibrate each instrument. The use of glass disks may avoid the potential quarantine issues required to bring a wheat calibration set into India and has an added benefit in the inherent stability and robustness of glass standards, whilst also eliminating the potential contamination, degradation and infestation that can occur when using plant material in calibrations.

## Materials and methods

### Samples

As previously reported (Paltridge et al., 2012a), whole grain wheat reference samples are not commercially available. Consequently, a set of calibration and validation samples for this study was obtained from a wheat biofortification breeding program at CIMMYT, Mexico. All samples were analyzed in duplicate by ICP-OES using a nitric acid/perchloric digestion method, at Waite Analytical Services, Adelaide, Australia (Wheal et al., 2011) to determine robust reference values. No samples contained > 4 mg Al kg^−1^, indicating they could be regarded as relatively free of soil contamination.

10 custom-made 40 mm diameter glass disks (FLUXANA® GmbH & Co. KG Borschelstr. 3, 47551 Bedburg-Hau, Germany) with a range of nominal Fe and Zn levels (Table 1) were tested in a preliminary study to determine how glass standards compare with XRF responses observed with wheat grain. An additional 10 glass disks (FLUXANA, Germany) were used for validation of this approach at 5 Indian laboratories.

**Table 1:** **Nominal elemental concentration of glass standards used for preliminary study**.

	**Nominal Zn concentration (mg kg^−1^)**	**Nominal Fe concentration (mg kg^−1^)**	**Matrix-adjusted Zn concentration (mg kg^−1^)**	**Matrix-adjusted Fe concentration (mg kg^−1^)**
Glass disk 1	2.5	2.5	4.2	17.4
Glass disk 2	5	5	6.9	17.7
Glass disk 3	7.5	7.5	9.2	19.2
Glass disk 4	10	10	12.8	20.1
Glass disk 5	25	25	29.0	26.6
Glass disk 6	50	50	52.0	38.1
Glass disk 7	75	75	84.6	50.1
Glass disk 8	100	100	112.3	53.6
Glass disk 9	150	150	173.1	82.8
Glass disk 10	200	200	268.6	107.5

*Matrix-adjusted values determined from the average of 5 replicate analyses of each glass disk with XRF wheat grain calibration*.

Twenty bread and durum wheat samples were used as the validation samples in this trial and analyzed with each of the XRFs in India and subsequently analyzed with ICP-OES in Australia (Wheal et al., 2011).

All plant samples were sterilized by gamma irradiation at 50 kGray (5 Mrad) prior to release into Australia for analysis.

### EDXRF

An Oxford Instruments X-Supreme 8000 fitted with a 10 place auto-sampler suitable for 40 mm Al cups was used for all XRF analyses. Measurement conditions are summarized in Table 2, as previously reported in the literature (Paltridge et al., 2012a). Wheat samples were analyzed in Al cups lined with 30 mm polypropylene inner cups sealed at one end with 4 μm Poly-4 XRF sample film (Oxford Instruments, UK). Whole grain analysis was performed with a minimum mass of 4 g wheat and a 60 s active scan time.

**Table 2:** **EDXRF conditions for the analysis of Zn and Fe**.

**Conditions**	**Zn**	**Fe**
Atmosphere	Air	Air
X-ray tube	Tungsten	Tungsten
Voltage	26 kV	15 kV
Current	115 μA	200 μA
Peak detected	Kα	Kα
Acquisition time	60 s	60 s
Tube filter	W5	A6
Detector	Silicon drift detector	Silicon drift detector

### Adjusted concentration determination for glass disks

Glass disks were scanned with XRF by placing the disk directly into the sample cup. Each disk was scanned 5 times with the existing grain calibration method (as per Paltridge et al., 2012a). The result of the 5 scans was averaged and this value used as the “adjusted” value for calibrations (Table 1).

### In-country validation

Each of the 5 XRFs had an updated glass method that was installed and calibrated. Additionally, the grain calibration developed at Flinders University (Paltridge et al., 2012a) was transferred electronically to each of the instruments. 20 bread and durum wheat samples were consequently scanned on each of the 5 XRFs with each of the 3 XRF calibration methods: glass calibration, electronic transfer calibration and the existing calibration method.

### Statistics

Statistical calculations used are defined below as per the literature (Perring and Andrey, 2003).

$$\begin{array}{cc} \text{Concentration determined by ICP-OES} & y_{i}\\ \text{Concentration determined by EDXRF} & {\hat{\text{y}}}_{\text{i}}\\ \text{Bias} & \frac{\sum_{i\;=\;1}^{n}\left({\hat{y}}_{i}\text{-}y_{i}\right)}{n}\\ \text{Standard error of prediction (SEP)} & \sqrt{\frac{\sum_{i\;=\;1}^{n}{\left({\hat{y}}_{i}\text{-}y_{i}\right)}^{2}}{n}} \end{array}$$

## Results

### Glass calibration validation

**Figure 1:**
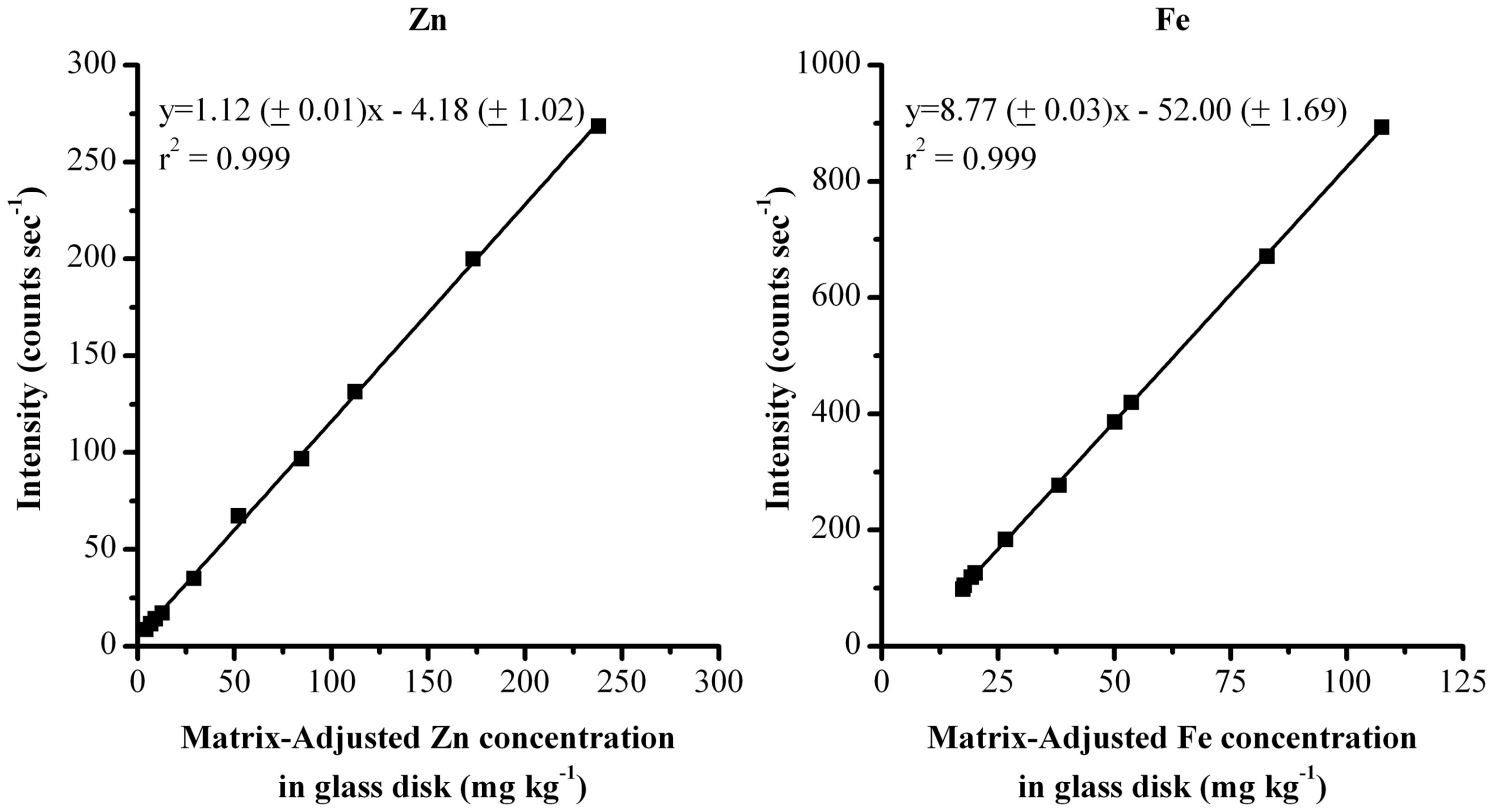
**XRF calibration for Zn and Fe utilizing adjusted reference values of 10 glass disks**.

Due to the difference in the wheat grain and glass disk matrices, each of the glass standards was measured 5 times with the wheat grain calibration method. The average value from these analyses was used to determine a matrix-adjusted value for the glass standards and account for the matrix difference. Absolute and adjusted Zn and Fe concentrations are reported in Table 1. The resulting adjusted glass calibrations are shown in Figure 1 (the initial grain calibration with *r*^2^ = 0.97 and 0.73 for Zn and Fe respectively can be seen in the literature, Paltridge et al., 2012a).

**Figure 2:**
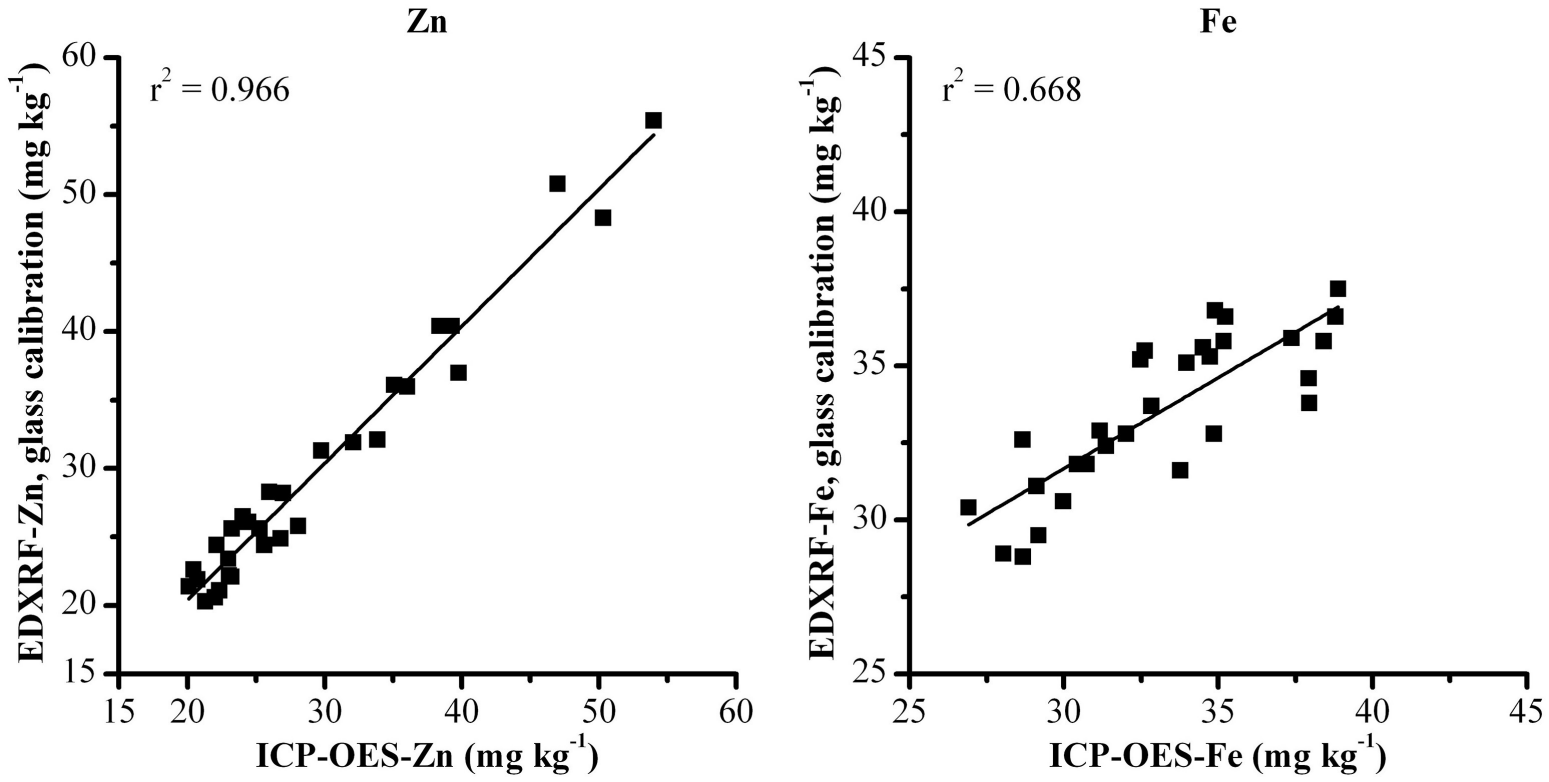
**Validation of glass calibration method for Zn and Fe analysis with 30 whole grain wheat samples analyzed with XRF and ICP-OES**.

In order to validate the glass calibration method, 30 wheat samples were analyzed via duplicate ICP-OES and consequently scanned with the glass calibration XRF method. The resulting validation results are shown in Figure 2. This shows a strong correlation between XRF and ICP-OES results with the glass calibration (*r*^2^ = 0.966 for Zn and *r*^2^ = 0.668 for Fe, as shown in Figure 2) and are comparable with the results from the grain calibration reported previously by Paltridge et al. (2012a) (Table 3).

**Table 3:** **Statistics for analytical methods for comparison of EDXRF analysis with validated ICP-OES analysis**.

**Statistic**	**EDXRF grain calibration^a^**		**EDXRF glass calibration**	
	**Zn**	**Fe**	**Zn**	**Fe**
*r*^2^	0.964	0.677	0.966	0.668
SEP	±1.92	±2.01	±1.75	±2.00
Bias^b^	0.828	0.440	0.368	0.406

a *, as in Paltridge et al. (2012a)*.

b *, XRF bias from ICP-OES not significantly different from zero at 95% level according to paired t-tests*.

**Figure 3:**
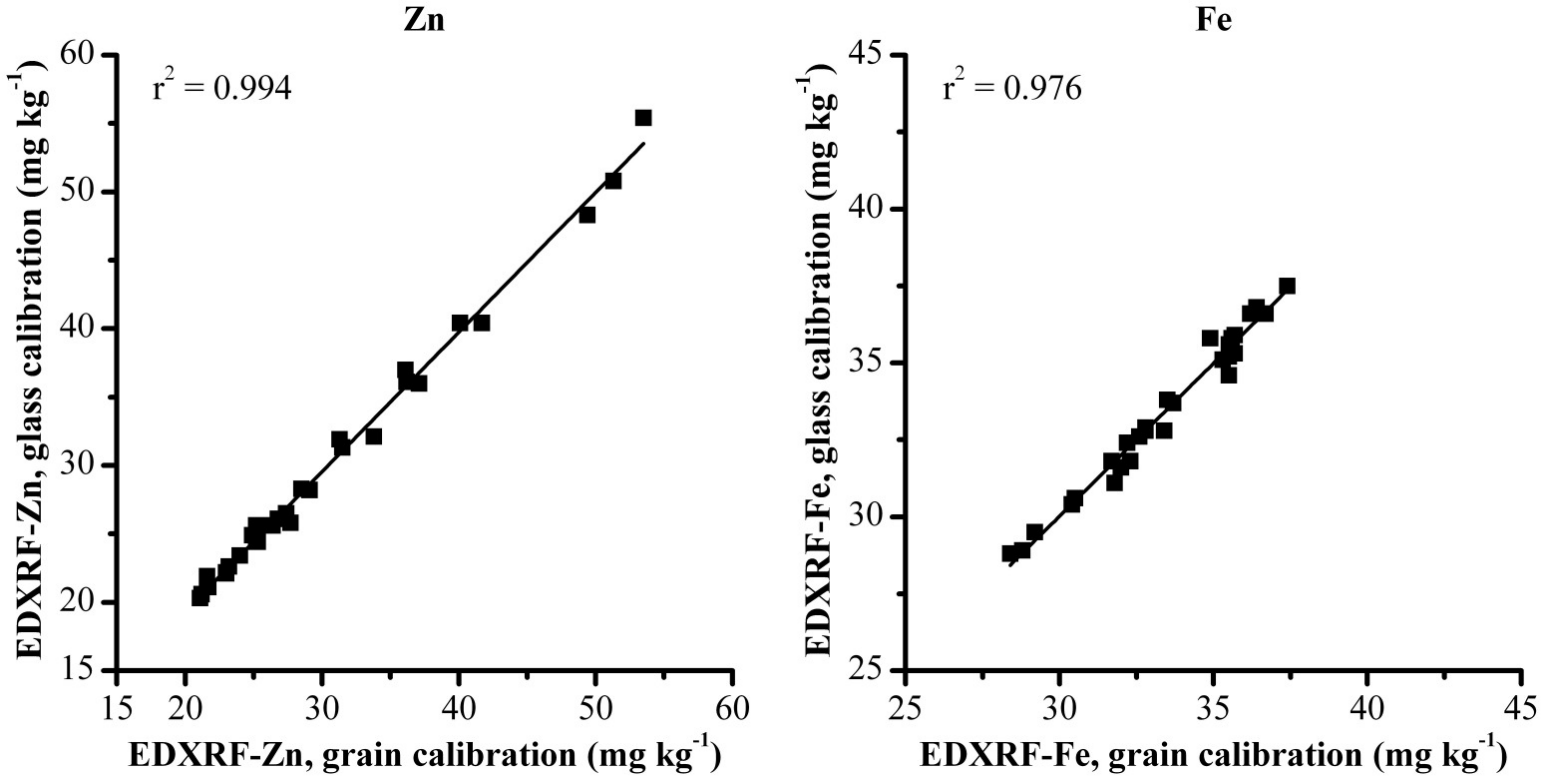
**Comparison of Zn and Fe analysis with glass and grain XRF calibration methods**.

These same samples were also analyzed with the previously validated grain XRF calibration to compare the effect of the glass calibration with the XRF grain calibration method (Figure 3). The comparison between XRF results from the two methods shows a strong correlation between both XRF calibration methods with *r*^2^ > 0.97 for both Zn and Fe.

The results from both the glass and grain calibrations when compared with ICP-OES reference values are shown in Table 3 and show both XRF methods produce results that strongly correlate with the ICP-OES reference values with SEP ± 2 mg kg^−1^ and bias of < 1 mg kg^−1^ for both Fe and Zn.

### In-country validation

Twenty bread and durum wheat samples were analyzed on each of the XRFs with three different calibration methods: glass, electronic transfer and original calibration methods. This approach aimed to compare the benefits of the glass calibration over the previously employed electronic transfer approach and the existing calibration (a combination of electronic transfer and grain calibrations with locally determined reference values).

As per the preliminary calibration and validation experiments, 10 glass standards were prepared for each of the XRF laboratories in India and scanned 5 times with the grain calibration method (as per Paltridge et al., 2012a) on the Flinders University XRF instrument. The average of 5 scans was used to determine an adjusted Zn and Fe concentration in the corresponding wheat glass calibrations. An example of the adjusted values is presented in Table 4.

**Table 4:** **Example of elemental concentrations of glass disk standards used for validation study showing Zn and Fe concentration in the glass disks and the adjusted value used for calibration determined from mean of 5 replicate analyses of each glass disks with original grain calibration**.

	**Nominal Zn concentration (mg kg^−1^)**	**Nominal Fe concentration (mg kg^−1^)**	**Matrix-adjusted Zn concentration (mg kg^−1^)**	**Matrix-adjusted Fe concentration (mg kg^−1^)**
Glass disk 11	5	5	7.5	18.9
Glass disk 12	10	10	13.4	20.1
Glass disk 13	15	15	18.6	22.0
Glass disk 14	20	20	25.0	25.2
Glass disk 15	30	30	36.4	29.2
Glass disk 16	40	40	47.9	33.7
Glass disk 17	50	50	58.2	37.3
Glass disk 18	75	75	87.4	48.4
Glass disk 19	100	100	123.3	62.2
Glass disk 20	125	125	150.8	73.9

**Figure 4:**
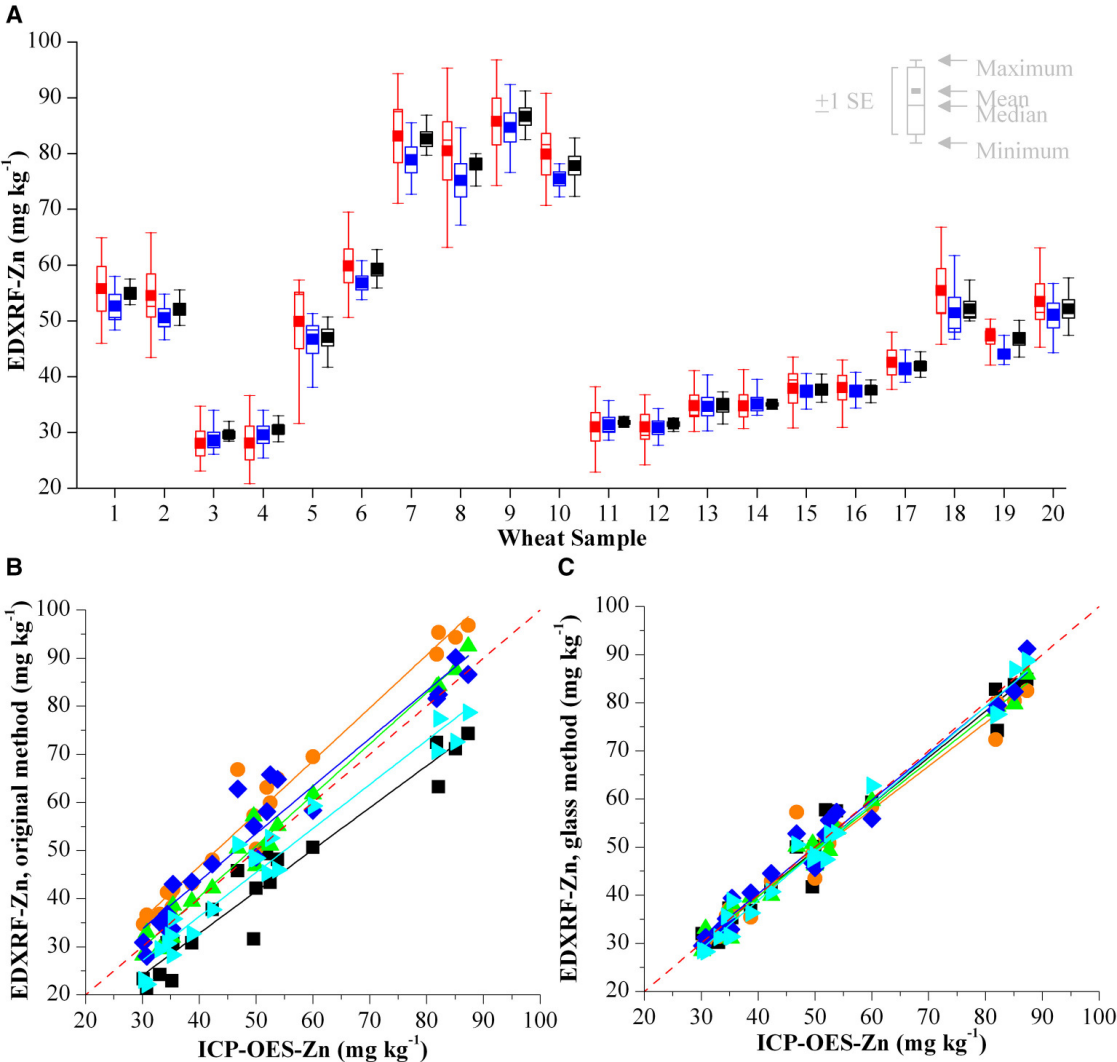
**(A)** Comparison of XRF Zn analysis of 20 wheat validation samples with three calibrations on 5 different HarvestPlus XRF instruments in India. Results from the original calibration are shown in red, the results from the electronic transfer method are shown in blue and the results from the glass calibration in black. Validation of **(B)** original XRF calibration method and **(C)** glass calibration method compared with the ICP-OES reference analysis for Zn. Results from each of the laboratories is represented with a different color and the red dashed line represents *y* = *x*.

**Figure 5:**
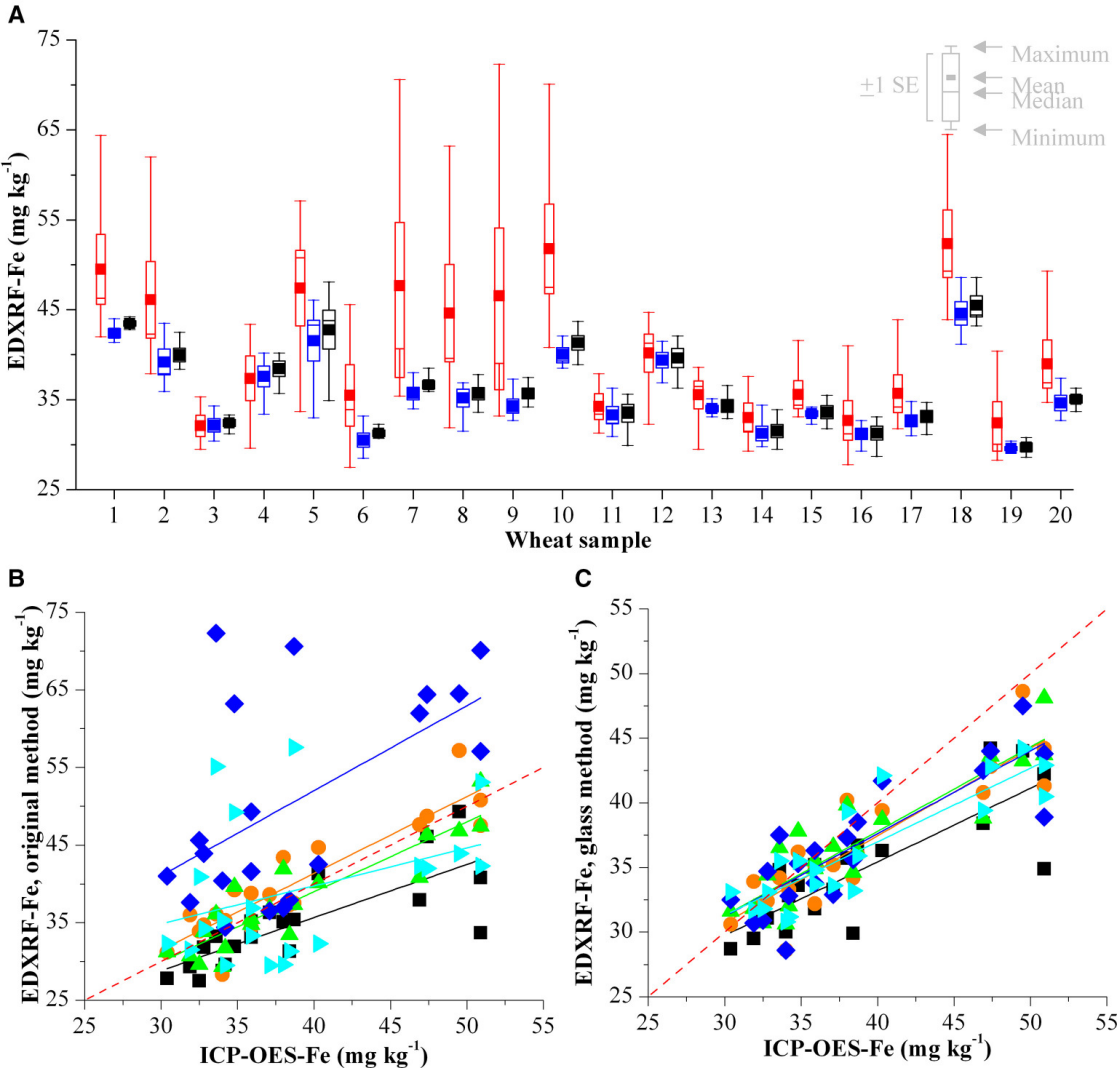
**(A)** Comparison of XRF Fe analysis of 20 wheat validation samples with three calibrations on 5 different HarvestPlus XRF instruments in India. Results from the original calibration are shown in red, the results from the electronic transfer method are shown in blue and the results from the glass calibration in black. Validation of **(B)** original XRF calibration method and **(C)** glass calibration method compared with the ICP-OES reference analysis for Fe. Results from each of the laboratories is represented with a different color and the red dashed line represents *y* = *x*.

The results of analysing 20 wheat samples on 5 different instruments with 3 different calibration methods is shown in Table 5. The difference between the XRF results for each sample is shown in Figures 4, 5, for Zn and Fe respectively. There is an improvement in both the accuracy of the XRF results when compared with ICP-OES (Table 6) and improvement in reproducibility between instruments when comparing the results with the glass calibration method (Table 5). Analysis of the same 20 wheat samples with the original calibration method on each of the 5 instruments resulted in a maximum difference between instruments of >30 mg kg^−1^ for both Zn and Fe with an average difference of ± 8.6 mg kg^−1^ and COV > 14% for both elements. Electronic transfer of a single calibration method improved the consistency between instruments slightly with a maximum difference of > 13 mg kg^−1^ for Zn and Fe (average difference of ± 4.6 mg kg^−1^ and ± 2.3 mg kg^−1^ respectively with COV >5%). Use of the glass calibration further improved the reproducibility between these results with a maximum difference of 11.8 mg kg^−1^ and 13.2 mg kg^−1^ and average of ± 2.8 mg kg^−1^ and ± 2.0 mg kg^−1^ for Zn and Fe results respectively and COV < 5%. The accuracy of the XRF results was also improved with the use of the glass calibration and individual laboratory results for each calibration method are shown in Table 6. The average XRF bias (when compared to ICP-OES reference analysis) ranged from 1.6 mg kg^−1^ and 2.3 mg kg^−1^ for Zn and Fe respectively for the glass calibration method. In comparison, the electronic transfer method resulted in a bias range of 7.5 mg kg^−1^ and 3.3 mg kg^−1^ for Zn and Fe whilst the original calibration methods resulted in even larger bias from ICP-OES analysis with 16.4 mg kg^−1^ and 15.8 mg kg^−1^.

**Table 5:** **Variation in XRF results between 5 instruments in India for analysis of 20 wheat validation samples for Zn and Fe, comparing the original methods, electronic transfer and glass calibrations**.

**Method**	**Average COV (%)**		**Maximum variation (mg kg**^**−1**^**)**		**Average variation (mg kg**^**−1**^**)**	
	**Zn**	**Fe**	**Zn**	**Fe**	**Zn**	**Fe**
Original calibration	14.6	17.4	32.1	39.1	8.6	8.6
Electronic transfer	8.2	5.3	17.4	13.1	4.6	2.3
Glass calibration	4.7	4.5	11.8	13.2	2.8	2.0

**Table 6:** **Correlation and average difference between ICP-OES and XRF results for Zn and Fe for 20 wheat validation samples reported for each of the 5 instruments trialed**.

		**Analysis method**	**Lab 1**	**Lab 2**	**Lab 3**	**Lab 4**	**Lab 5**
Correlation	Zn	Glass calibration	0.984	0.980	0.993	0.987	0.991
		Electronic transfer	0.977	0.981	0.987	0.982	0.991
		Original method	0.971	0.984	0.992	0.963	0.976
	Fe	Glass calibration	0.824	0.891	0.901	0.848	0.870
		Electronic transfer	0.787	0.892	0.894	0.853	0.869
		Original method	0.776	0.906	0.906	0.552	0.366
Average bias (mg kg^−1^)	Zn	Glass calibration	−0.661	−1.271	−1.001	0.019	−1.021
		Electronic transfer	−4.966	2.499	−3.116	−0.471	−4.436
		Original method	−8.616	7.749	0.999	3.489	−4.641
	Fe	Glass calibration	−4.010	−2.125	−1.750	−1.915	−2.450
		Electronic transfer	−5.030	−1.725	−3.290	−1.765	−3.525
		Original method	−3.935	1.355	−0.895	11.885	0.400

## Discussion

**Figure 6:**
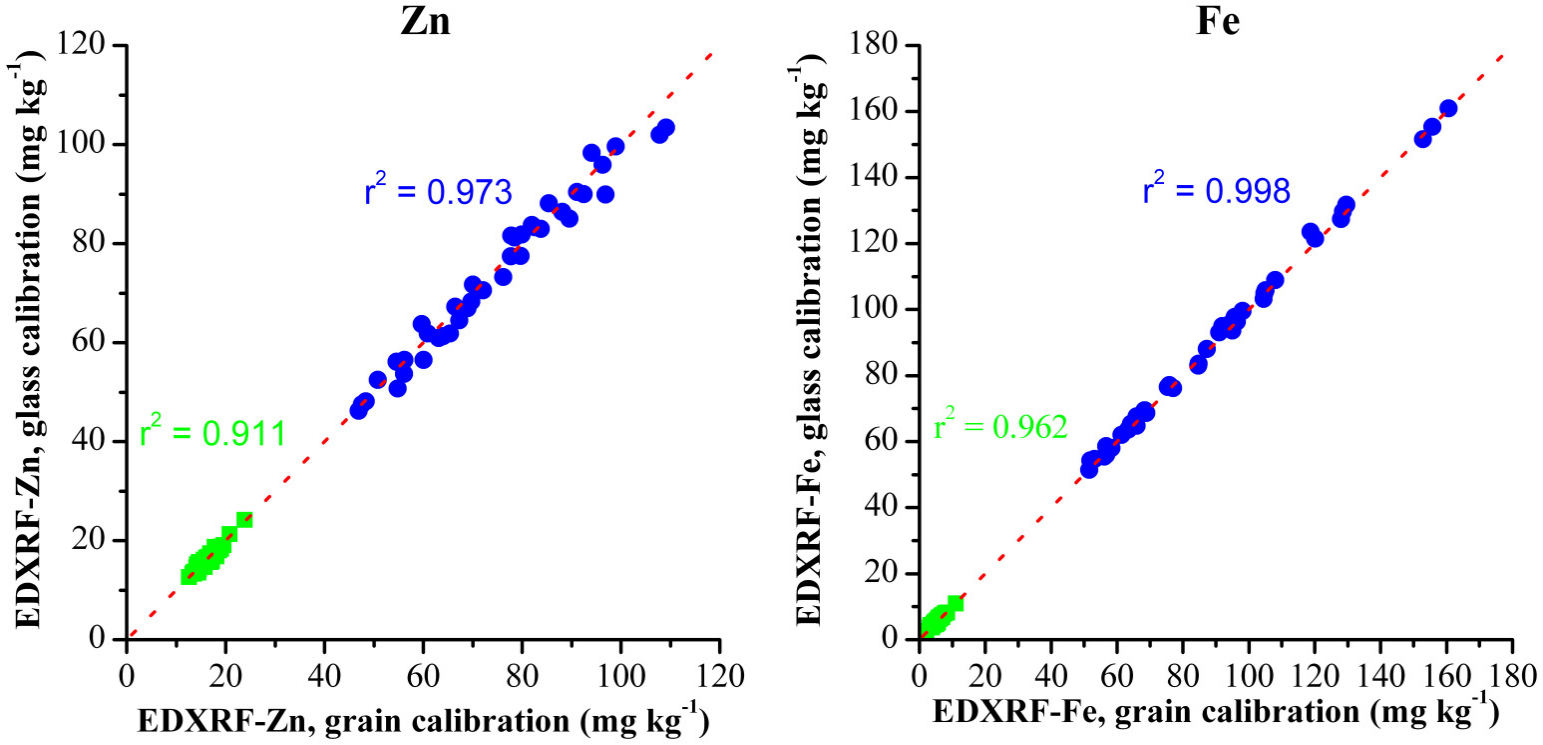
**Validation of glass calibration for screening Fe and Zn in whole grain rice (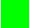) and pearl millet (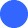) and *y* = *x* represented by the red dashed line**.

Within the HarvestPlus wheat biofortification program, the primary objective is to develop wheat varieties with high Zn levels and with elevated Fe a secondary trait. We have previously shown the benefits of XRF as a high throughput and cost effective method of screening for high levels of these micronutrients in conventional plant breeding. The success of this technique has been evident with 9 XRFs used for screening wheat within the HarvestPlus program. XRF analysis has various benefits over conventional ICP-OES analysis. Sample throughput with XRF is rapid with 10 samples able to be analyzed in less than 60 min with little to no sample preparation required. This is a significant benefit over ICP-OES analysis, which requires samples to be ground and digested prior to analysis (Wheal et al., 2011). Additionally, ICP-OES analysis is carried out in specialized laboratories which are often not locally available to plant breeders and consequently requires samples be sent abroad which is both expensive and time consuming. Furthermore, there is a significant saving when analysing hundreds of samples from a breeding trial with XRF. This analysis cost is approximately 80% less than the cost for sending samples for ICP analysis (AUD $ 5.00 for XRF analysis in comparison to AUD $ 25.00 for ICP-OES analysis). When considering the additional cost of the glass disks (€90 each) this is not a significant outlay as these are stable for long periods without the risk of degradation or infestation, which can occur when using grains. The homogeneity of these glass disks reduces the number of samples required for a calibration when compared to a traditional grain calibration (10 glass disks compared with 20+ grain samples) and transporting 10 glass disks internationally is more convenient than the quarantine difficulties associated with transporting whole grains. Additionally, glass disks can be used to calibrate for multiple crops, further improving the cost-benefit of these standards. It is possible to use the same process discussed above to produce an XRF calibration for other crops analyzed with XRF within the HarvestPlus program including rice and pearl millet. The validation of 25 rice and pearl millet samples is shown in Figure 6 and resulted in equivalent validation results to that of the reported grain calibrations in the literature (Paltridge et al., 2012b).

One of the challenges faced is ensuring accurate calibrations on the individual XRF instruments. Errors in the calibration will not significantly affect individual breeding programs, as screening will still be possible to identify those genotypes that are high in Zn and Fe. However, when comparing results across different laboratories, these errors could have a significant effect when evaluating breeding materials. We have shown here the previous approaches for calibrating instruments with either electronic transfer of the calibration method or the use of locally sourced calibration reference material are not ideal and this highlights the need for a course of action to improve reproducibility and accuracy across the suite of HarvestPlus instruments. The variation between analyses from different XRF instruments cannot be accounted for with the electronic transfer approach. This is shown in the *in country validation* study from Figures 4, 5 with up to 17.4 and 13.1 mg kg^−1^ difference for Zn and Fe analysis of the same samples on different instruments. We have also demonstrated that the results from analyses using the original calibrations that show significant differences when analysing the same samples. This is likely due to the combination of instrumental variation and poor quality reference analysis of locally provided calibration samples. The implementation of non-matrix matched glass disks for XRF calibration has significantly improved the reproducibility between analyses on different instruments, with the average variation between analyses is < 5%. Considering the non-homogenous nature of the whole grain samples, this is deemed as suitable for use with high throughput screening within the HarvestPlus program. Furthermore the use of the glass calibration improved the accuracy of the XRF analysis with the average results within ~2 mg kg^−1^ of ICP-OES analysis.

Difference between XRF and ICP-OES results is expected due to the inherent variability caused by sampling whole grain and the nature of XRF analysis. Sample reproducibility could be improved with longer scan times, replicate analyses and grinding and pressing samples for XRF analysis. However, as the aim of this method is for high throughput screening, it has been concluded that single replicate analysis is suitable for this application (Paltridge et al., 2012a). Furthermore, the validation results of the glass calibration indicate the results from this are comparable with both the ICP-OES reference values (*r*^2^ = 0.965 and 0.668 for Zn and Fe respectively, Figure 2).

The Fe results from the in-country validation do not correlate as closely with ICP-OES as the Zn results; this is particularly evident with samples containing high concentrations of Fe. This is likely to be due to multiple factors including the calibration. As discussed previously, the XRF signal for Fe is not as intense as with heavier elements (i.e., Zn). Additionally, the range of Fe concentrations available in the calibration grain samples is 26.1–41.2 mg kg^−1^, however, ICP-OES analysis of the wheat validation samples from India show that some of these samples have Fe concentrations over 50 mg kg^−1^ and as these are well above the highest calibration grain sample this is a likely cause of errors in analysis of samples with high Fe levels. As with all crops, it will be important to improve or adjust the grain calibrations as samples with higher levels of Fe and Zn have to be expected within the biofortification plant breeding programs. This is particularly important for Fe as the current low calibration range coupled with the lower XRF intensity for Fe means any increase in the strength of the calibration could improve the XRF accuracy for Fe significantly. Another possible cause of the errors in the Fe analysis could be due to the presence of soil contamination. According to HarvestPlus standards, grain samples with Al > 4 mg kg^−1^ are considered as having significant soil contamination (Pfeiffer and McClafferty, 2007b; Yasmin et al., 2014). Soil contamination is more likely to affect the Fe analysis of the sample rather than Zn (Sillanpää, 1982), consequently the presence of high levels of Al is an indicator of high levels of soil contamination which can complicate the XRF analysis. ICP-OES analysis of the validation samples shows high levels of Al (Supplemental Table 1), which can be attributed to soil/dust contamination of the samples. XRF is calibrated with clean wheat samples and the presence of Fe (due to dust/soil) is a likely cause for Fe inaccuracies between the ICP-OES and XRF results, as shown in Figure 5. This contamination is further complicated by the fact that the 20 validation samples were analyzed with XRF (on 5 different instruments) and subsequently analyzed with ICP-OES. Consequently it is not possible to determine if this contamination was present in the samples from the initial analysis or if this occurred during transit, handling and/or analysis at the various XRF sites.

## Conclusions

EDXRF has been highly successful within the HarvestPlus program and enabled rapid and cost-effective screening of thousands of wheat samples. Many of the HarvestPlus XRFs have been calibrated via the electronic transfer of a previously developed whole grain calibration. This method is not able to account for slight instrumental differences; consequently we have investigated the use of non-matrix matched glass disks to calibrate 5 XRF instruments in India for wheat analysis. Using this approach it was possible to reduce the contamination and to overcome quarantine issues associated with international shipping of plant calibration material. Additionally the use of glass standards to calibrate the XRFs resulted in an improvement in the reproducibility to less than 5% variability between analyses and improves the accuracy of analysis significantly. Our results emphasize the benefits of using non-matrix matched calibration standards to improve accuracy and reproducibility between instruments globally across the HarvestPlus program. This is not limited to wheat, but is also applicable for other staple food crops within the biofortification breeding programs around the world.

## Author contributions

GG and JS contributed to experimental design along with acquisition, analysis, and interpretation of the data and drafting the manuscript.

### Conflict of interest statement

The authors declare that the research was conducted in the absence of any commercial or financial relationships that could be construed as a potential conflict of interest.
