# Supplementary material for: Non-matrix Matched Glass Disk Calibration Standards Improve XRF Micronutrient Analysis of Wheat Grain across Five Laboratories in India
Source: Front Plant Sci. 2016 Jun 8;7:784. doi: 10.3389/fpls.2016.00784 (PMC4896964; doi:10.3389/fpls.2016.00784)
Supplement: Supplementary file 1 [file Table1.docx]

Supplemental Table 1: Fe, Zn and Al concentration (mg kg^-1^) in 20 wheat validation samples from India

| Sample | Zn (mg kg^-1^) | Fe (mg kg^-1^) | Al (mg kg^-1^) |
| --- | --- | --- | --- |
| 1 | 54 | 47 | 6.6 |
| 2 | 53 | 47 | 4.5 |
| 3 | 30 | 34 | 6.7 |
| 4 | 31 | 38 | 17.0 |
| 5 | 50 | 51 | 4.5 |
| 6 | 60 | 32 | 6.7 |
| 7 | 85 | 39 | 6.1 |
| 8 | 82 | 35 | 5.1 |
| 9 | 87 | 34 | 4.6 |
| 10 | 82 | 51 | 4.6 |
| 11 | 35 | 38 | 7.2 |
| 12 | 33 | 40 | 10.0 |
| 13 | 35 | 37 | 4.3 |
| 14 | 34 | 32 | 3.8 |
| 15 | 39 | 36 | 4.9 |
| 16 | 35 | 30 | 4.0 |
| 17 | 42 | 33 | 4.7 |
| 18 | 47 | 50 | 3.5 |
| 19 | 50 | 34 | 3.7 |
| 20 | 52 | 36 | 5.3 |
